# Effectiveness of drug-loaded poly(ethylene glycol) and poly(lactic-co-glycolic-acid) nanoparticles in the *in vitro* treatment of breast cancer: a systematic review

**DOI:** 10.3389/fphar.2025.1710176

**Published:** 2026-01-05

**Authors:** Cristian Sandoval-Vásquez, Isabella Cárcamo, Paula Lagos, Anaís Muñoz, Francisco Zavala, Valentina Colil, Francisca Villagrán-Silva, Edgar Vásquez-Carrasco, Jordan Hernandez-Martinez, Pablo Valdés-Badilla, Francisco Torrens, Paola Fincheira, Paulina Sepúlveda

**Affiliations:** 1 Escuela de Tecnología Médica, Facultad de Salud, Universidad Santo Tomás, Los Carreras, Osorno, Chile; 2 Departamento de Medicina Interna, Facultad de Medicina, Universidad de La Frontera, Temuco, Chile; 3 Carrera de Tecnología Médica, Facultad de Medicina, Universidad de La Frontera, Temuco, Chile; 4 Programa de Doctorado en Ciencias Morfológicas, Facultad de Medicina, Universidad de La Frontera, Temuco, Chile; 5 School of Occupational Therapy, Faculty of Psychology, Universidad de Talca, Talca, Chile; 6 Centro de Investigación en Ciencias Cognitivas, Faculty of Psychology, Universidad de Talca, Talca, Chile; 7 VITALIS Longevity Center, Universidad de Talca, Talca, Chile; 8 Department of Physical Activity Sciences, Universidad de Los Lagos, Osorno, Chile; 9 Department of Education, Faculty of Humanities, Universidad de la Serena, La Serena, Chile; 10 Department of Physical Activity Sciences, Faculty of Education Sciences, Universidad Católica del Maule, Talca, Chile; 11 Sports Coach Career, Faculty of Life Sciences, Universidad Viña del Mar, Viña del Mar, Chile; 12 Institut Universitari de Ciència Molecular, Universitat de València, València, Spain; 13 Departamento de Ciencias Preclínicas, Facultad de Medicina, Universidad de La Frontera, Temuco, Chile; 14 Departamento de Ciencias Básicas, Facultad de Medicina, Universidad de La Frontera, Temuco, Chile

**Keywords:** breast cancer, PEG–PLGA nanoparticles, drug-delivery systems, antineoplastic agents, theranostics

## Abstract

**Background:**

Breast cancer treatment remains a major challenge to modern medicine and has driven the need for nanotechnology-based strategies to improve drug delivery and overcome chemoresistance. Poly(ethylene glycol) and poly (lactic-co-glycolic acid) (PEG–PLGA) nanoparticles (NPs) are a type of FDA-approved biodegradable copolymer (lactic + glycolic acids) that degrades into non-toxic metabolites (lactic acid and glycolic acid); it has emerged as a promising drug carrier owing to its biocompatibility, sustained release properties, and ability to enhance the cellular uptake of chemotherapeutic agents. This systematic review examines the efficacies of PEG–PLGA nanoparticles loaded with antineoplastic drugs on *in vitro* models of breast cancer cell lines.

**Methods:**

Following PRISMA guidelines, we conducted a comprehensive search of the Web of Science, Embase, MEDLINE, and Scopus databases to identify experimental studies published between 2014 and August 2025 that evaluated PEG–PLGA formulations applied to breast cancer cell lines. The methodological quality of each study was appraised using the National Institute for Health and Care Excellence (NICE) criteria.

**Results:**

Thirteen studies were chosen based on our inclusion criteria. Here, the PEG–PLGA nanoparticles were predominantly spherical (30–210 nm) and exhibited controlled release kinetics. Compared with free drugs, the nanoformulations significantly reduced cell viability, increased apoptosis, and induced cell-cycle arrest. Functionalization with ligands such as folic acid enhanced drug targeting and cytotoxicity, while the molecular analyses revealed upregulation of p53, Bax, and caspases as well as downregulation of *Bcl-2* and *hTERT* genes.

**Conclusion:**

PEG–PLGA nanoparticles can substantially improve the selectivity, bioavailability, and cytotoxic efficacies of anticancer drugs in breast cancer *in vitro*. These findings underscore their translational potential as next-generation drug-delivery systems, warranting *in vivo* validation as well as development of theranostic- and stimulus-responsive designs for personalized oncology.

**Systematic Review Registration:**

https://www.crd.york.ac.uk/PROSPERO/view/CRD420251076570.

## Graphical Abstract

**Figure:**
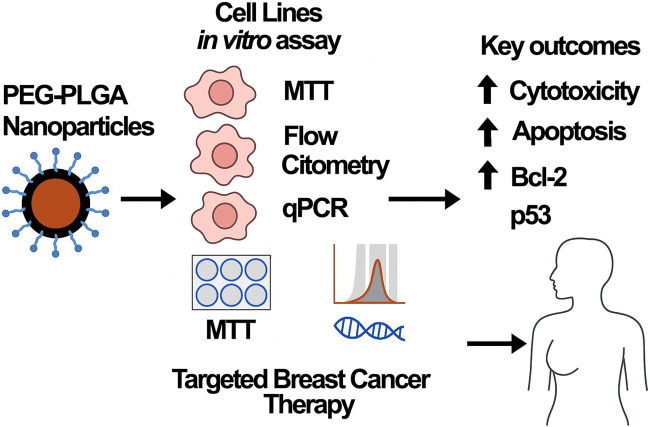

## 1 Introduction

### 1.1 Breast cancer burden and challenges

Breast cancer is the second most-prevalent malignancy globally, surpassed only by lung cancer, and remains as the leading cause of cancer-related mortality among women (Sung et al., 2021). Its etiology is multifactorial and involves demographic, genetic, hormonal, reproductive, and lifestyle-related risk factors, including age, female sex, obesity, family history, and unhealthy behaviors (Colditz and Bohlke, 2014; Key et al., 2001). Clinically, this condition often manifests as a palpable breast mass that is either self-detected or identified during routine examinations. Suspected cases of cancerous breast masses are often confirmed diagnostically through imaging techniques such as mammography and ultrasonography, followed by histopathological evaluation through biopsy (D’Orsi et al., 2013). Most breast carcinomas originate from the ductal or lobular epithelium and are categorized as carcinoma *in situ* or invasive carcinomas. Carcinoma *in situ* is confined within the ducts or lobules without breaching the basement membrane, whereas invasive carcinomas infiltrate the stroma and surrounding tissues (Schnitt, 2010). The most frequent histological subtypes are invasive ductal carcinoma and invasive lobular carcinoma (Lakhani et al., 2012). Therapeutic strategies against these carcinomas depend on the stage of the disease and may include surgery, radiotherapy, chemotherapy, endocrine therapy, or targeted agents. In advanced or metastatic settings, the treatment is mainly palliative, with the aim of extending survival and preserving the quality of life (Cardoso et al., 2020). Despite numerous therapeutic advances in recent times, challenges persist with respect to systemic toxicity, multidrug resistance, and suboptimal tumor bioavailability, underscoring the need for more effective drug-delivery strategies.

### 1.2 Nanoparticles in drug delivery

Nanotechnology has emerged as a transformative platform to enhance the pharmacokinetic and pharmacodynamic profiles of anticancer agents. Among the various polymers investigated in related literature, poly(lactic-co-glycolic acid) (PLGA) nanoparticles (NPs) have been extensively employed in drug delivery owing to their favorable biocompatibility and biodegradability profiles (Danhier et al., 2012). However, their rapid clearance via plasma protein opsonization and recognition by the reticuloendothelial system have limited clinical translation (Owens and Peppas, 2006). Among the various fabrication techniques available, nanoprecipitation is widely used because of its simplicity; this process involves dissolving the polymer in an organic solvent (e.g., acetone), introducing the solution into an aqueous medium, allowing spontaneous self-assembly, and removing the solvent subsequently (Fessi et al., 1989). In oncology, poly(ethylene glycol) with PLGA (PEG–PLGA) NPs offer a promising alternative to conventional chemotherapy by enabling controlled and targeted drug release, thereby improving the therapeutic efficacy while minimizing systemic toxicity and drug resistance (Shi et al., 2010; Parveen et al., 2012).

### 1.3 PEG–PLGA NPs: advantages and limitations

Surface modification of the PLGA NPs with PEG was developed to address issues related to immunogenicity and rapid clearance. PEGylation reduces immunogenicity by shielding the antigenic sites, enhances NP hydrophilicity and stability, and prolongs systemic circulation (Jokerst et al., 2011; Makadia and Siegel, 2011). Structurally, PEG–PLGA NPs exhibit a core-shell configuration, in which the PLGA core encapsulates therapeutic molecules, while the PEG shell provides protection against immune detection (Mu and Feng, 2003). Despite these advantages, the PEG–PLGA NPs have some critical limitations: i) the effectiveness of PEGylation depends on the chain density and length, which influence protein corona formation and biodistribution; ii) repeated administration may induce accelerated blood clearance or anti-PEG immune responses; iii) physicochemical characterizations of the particles are often incomplete, along with limited knowledge regarding the zeta potential, polydispersity, and long-term stability under physiologically relevant conditions; iv) ligand functionalization may improve active targeting but can compromise the stealth effects of PEG, increase formulation complexity, and limit scalability. Therefore, we aimed to assess the effectiveness of drug-loaded PEG–PLGA NPs in the treatment of breast cancer based on *in vitro* studies reported over the last decade.

## 2 Methods

### 2.1 Protocol and registration

We conducted a systematic evaluation of quantitative studies to assess the effectiveness of drug-loaded PEG-PLGA NPs in breast cancer therapy by focusing exclusively on *in vitro* experimental models. Our review was conducted in accordance with the preferred reporting items for systematic reviews and meta-analyses (PRISMA) guidelines (Page et al., 2021). The study protocol was prospectively registered with the PROSPERO database (CRD420251076570) (Vásquez and Sandoval, 2025).

### 2.2 Search strategy and selection criteria

#### 2.2.1 Search strategy

A comprehensive literature search was conducted across four electronic databases, namely, MEDLINE, Embase, Scopus, and Web of Science, covering all available records up to August 2025. The search was designed to identify peer-reviewed *in vitro* studies without restrictions on language or publication date. The exclusions included conference abstracts, books, book chapters, editorials, letters to the editor, protocol records, reviews, case reports, and reviews or *in vivo* studies given the purpose of this review, which is to synthesize the mechanistic and cytotoxic effects of PEG–PLGA drug-loaded NPs under controlled *in vitro* conditions independent of the confounding physiological variables (e.g., metabolism, immune clearance, and vascular barriers) present in *in vivo* models. The inclusion of studies was guided by the population, intervention, comparator, outcome, and study design (PICOS) framework, as outlined in Table 1.

**TABLE 1:** PICOS criteria used in the systematic review.

Criterion	Inclusion criterion	Exclusion criterion
Population	Human breast cancer cell lines (MCF-7, MDA-MB-231, T47D, MCF-10A, MDA-MB-436, or SKBR3)	Non-breast-cancer models; *in vivo* animal studies; clinical trials
Intervention	Drug-loaded PEG–PLGA nanoparticles (including functionalized forms such as folic-acid- or peptide-conjugated and dual-drug systems)	Nanoparticles not based on PEG–PLGA; unloaded (empty) nanoparticles
Comparison	Reported comparisons with free (non-encapsulated) drugs or non-targeted nanoparticles (e.g., PLGA without PEGylation or ligand functionalization)	Studies without a comparator group
Outcomes	Availability of quantitative physicochemical or biological outcome, such as particle size, zeta potential, morphology, encapsulation efficiency, release profile, cellular uptake, cytotoxicity (MTT/MTS assays), apoptosis/gene expression (p53, Bax, caspases, Bcl-2, and hTERT), cell-cycle arrest, and safety in non-tumorigenic cell lines	Studies lacking quantitative efficacy or mechanistic outcomes; articles not reporting nanoparticle performances in breast cancer models
Study design	*In vitro* experimental studies published in English between January 2014 and August 2025	Reviews, meta-analyses, editorials, commentaries, book chapters, conference abstracts, and non-English publications

Bax, Bcl-2-associated X protein (proapoptotic regulator); Bcl-2, B-cell lymphoma 2 (antiapoptotic regulator); hTERT, human telomerase reverse transcriptase; MTT, 3-(4,5-dimethylthiazol-2-yl)-2,5-diphenyltetrazolium bromide (cell viability assay); MTS, 3-(4,5-dimethylthiazol-2-yl)-5-(3-carboxymethoxyphenyl)-2-(4-sulfophenyl)-2H-tetrazolium (cell viability assay); p53, tumor protein p53 (tumor suppressor gene); PEG–PLGA, poly(ethylene glycol) with poly(lactic-co-glycolic acid).

**FIGURE 1:**
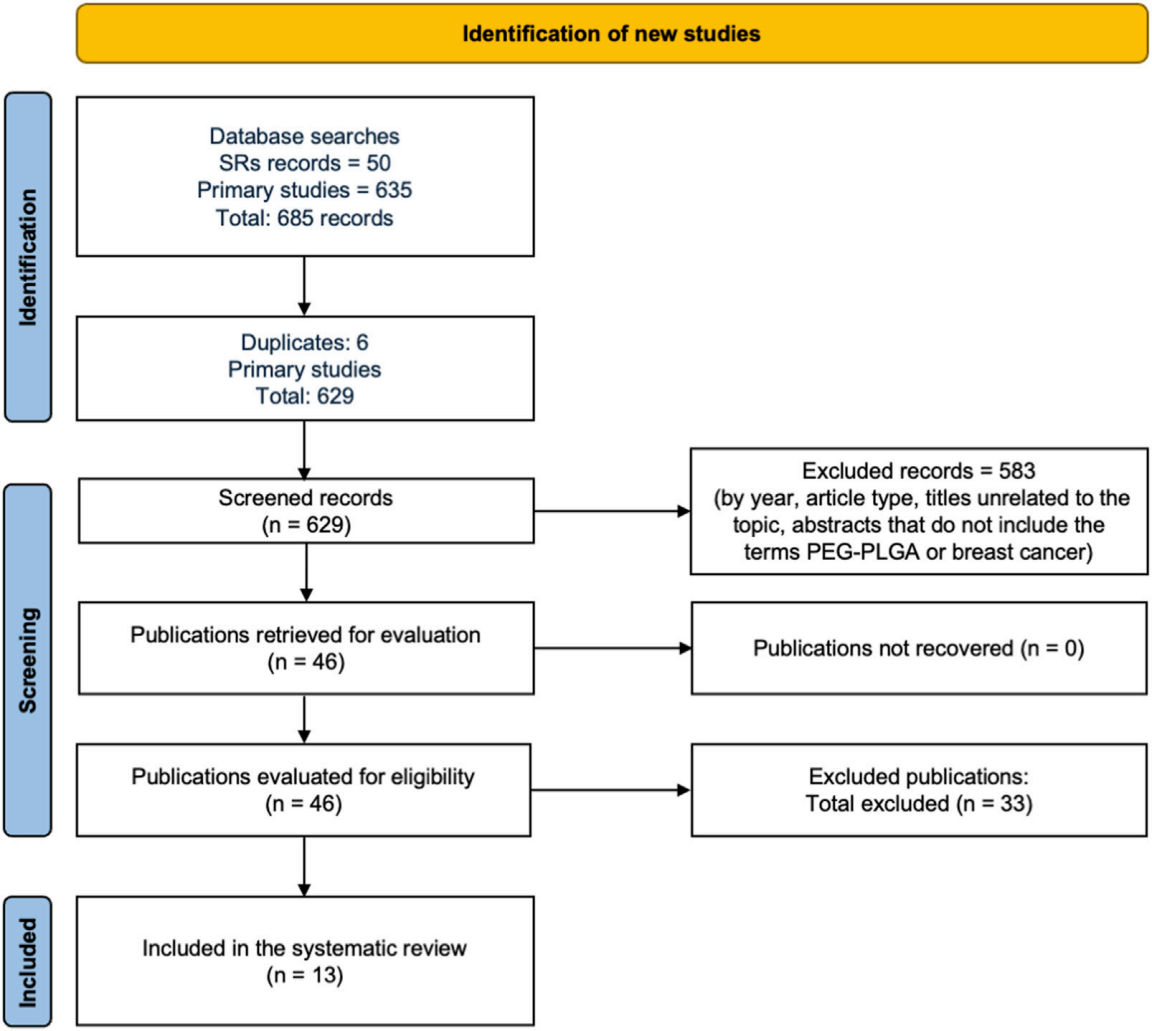
Flowchart of the systematic review.

The search strategy involved a combination of medical subject headings (MeSH) and free-text terms related to drug-loaded systems, PEG–PLGA NPs, breast cancer, and treatment. The Boolean search strings used were as follows: (“PEG–PLGA” OR “PLGA-PEG” OR “Poly (lactic-co-glycolic acid)” OR “PLGA nanoparticles” OR “PEGylated PLGA”) AND (“drug-loaded” OR “drug delivery system” OR “nanocarrier” OR “controlled release” OR “targeted delivery”) AND (“nanoparticles” OR “nanomedicine” OR “nanosystem”) AND (“cancer” OR “neoplasm” OR “tumor” OR “carcinoma” OR “oncology” OR “breast cancer” OR “mammary carcinoma”) AND (“treatment” OR “therapy” OR “therapeutic efficacy”). The lists of references of eligible studies and relevant reviews were manually screened to ensure comprehensive coverage, and only original studies that reported primary quantitative data were considered. The search and selection process implemented is summarized in the PRISMA flow diagram shown in Figure 1.

#### 2.2.2 Identification of relevant studies

An independent expert was consulted on the included articles and the inclusion and exclusion criteria to help find more relevant studies. The chosen expert had to meet two established requirements, namely, hold a PhD in health sciences and have peer-reviewed works published in journals with an impact factor, in accordance with the Journal Citation Reports® on drug-loaded treatments, NPs, and breast cancer. Our search approach was not revealed to the expert so as to avoid bias in their searches. After completing these procedures, on 30 August 2025, we searched the database to find relevant retractions or errata related to the included articles.

#### 2.2.3 Types of study and design

The studies included herein were used to investigate the effectiveness of drug-loaded PEG–PLGA NPs in breast cancer therapy and the impacts of PEG–PLGA NPs on various biological pathways. Furthermore, we deemed it essential that these investigations were conducted in the English language. The inclusion criteria for the studies considered are as follows: (1) the research included studies focusing on the collection of numerical data or a combination of both numerical and qualitative data; (2) the statistical analysis techniques used in the research included both descriptive and inferential methods involving parametric and non-parametric approaches; (3) the types of studies included in the research were randomized controlled trials, cross-sectional studies, experimental research, and clinical trials. The exclusion criteria for the studies considered are as follows: (1) there was lack of quantitative data or specific numerical values in the studies; (2) the investigations were not reported in peer-reviewed scholarly publications; (3) the sources included conference abstracts, systematic reviews, or letters to the editor; (4) the studies did not primarily focus on investigating the use of drug-loaded PEG–PLGA NPs in breast cancer treatment or provide comprehensive descriptions of the anti-inflammatory activities and/or excluded anti-oncogenic qualities.

#### 2.2.4 Cell lines

For the *in vitro* studies, breast cancer cell lines including MCF-7, MDA-MB-231, T47D, MCF-10A, MDA-MB-436, and SKBR3 were analyzed. These models were employed to evaluate the anticancer efficacies of the PEG–PLGA NPs, particularly their effects on the anti-inflammatory activities and/or anti-oncogenic qualities.

#### 2.2.5 Quality assessment and risk of bias

The methodological quality of each quantitative study was initially assessed by a single reviewer based on the National Institute for Health and Care Excellence (NICE) rating methodology (National Institute for Health and Care Excellence, 2012). Then, a second reviewer autonomously confirmed the precision of the evaluations. All differences were addressed through dialogue and agreement. None of the studies were rejected on the grounds of problems related to methodological quality.

#### 2.2.6 Data extraction and synthesis

Two independent reviewers assessed the titles, abstracts, and full texts of all the retrieved records to confirm eligibility. Each reviewer prepared the summary tables and flow diagrams aligned with their search outcomes. The extracted data were then compared, and discrepancies were resolved with the involvement of a third reviewer, who carefully re-examined the textual content, tables, and figures of the included studies. If any disagreements remained unresolved, the original study authors were contacted for clarification. Additionally, two researchers independently reviewed the results and discussion sections of eligible studies to determine the therapeutic roles of drug-loaded PEG–PLGA NPs in cancer treatment and their influences on different biological pathways. The synthesis focused on parameters related to the efficacy of PEG–PLGA NPs in cancer progression based exclusively on *in vitro* experimental models. The search procedures were in accordance with the PRISMA guidelines, as presented in Figure 1.

## 3 Results

### 3.1 Study selection

A total of 635 original studies were identified through the database search, and six studies were excluded from this list owing to duplication. Of the remaining 629 records, 583 were excluded on the basis of publication year, article type, titles unrelated to the topic, and abstracts that did not include the terms PEG–PLGA or breast cancer. Following a full-text review of the remaining 46 works, 33 studies were excluded for not matching the required study type. Ultimately, 13 studies were included in the systematic review described herein (Esim et al., 2024; Jafari-Gharabaghlou et al., 2023; Farajzadeh et al., 2018; Yildiz et al., 2018; Haggag et al., 2020; El-Hammadi et al., 2017; Kordi et al., 2016; Singh et al., 2015; Houdaihed et al., 2020; Sharma et al., 2018; Javan et al., 2019; Tabatabaei Mirakabad et al., 2016; Gorjikhah et al., 2017) (Figure 1).

### 3.2 Summary of the included studies

The 13 experimental studies shortlisted herein all examined PEG–PLGA-based polymeric NPs encapsulating antineoplastic agents for *in vitro* testing on human breast cancer cell lines. These studies were conducted across multiple countries, namely, Turkey (Esim et al., 2024), Iran (Jafari-Gharabaghlou et al., 2023; Farajzadeh et al., 2018; Kordi et al., 2016; Javan et al., 2019; Tabatabaei Mirakabad et al., 2016; Gorjikhah et al., 2017), United States (Yildiz et al., 2018), United Kingdom (Haggag et al., 2020; Sharma et al., 2018), Canada (Houdaihed et al., 2020), Spain (El-Hammadi et al., 2017), and India (Singh et al., 2015). The main cell models used in these studies were MCF-7 (Esim et al., 2024; Haggag et al., 2020; El-Hammadi et al., 2017; Singh et al., 2015; Houdaihed et al., 2020; Tabatabaei Mirakabad et al., 2016), MDA-MB-231 (Esim et al., 2024; Jafari-Gharabaghlou et al., 2023; Yildiz et al., 2018; Houdaihed et al., 2020; Sharma et al., 2018; Javan et al., 2019), and T47D (Farajzadeh et al., 2018; Kordi et al., 2016; Gorjikhah et al., 2017); additional cell lines such as SKBR3 and MDA-MB-436 were also tested in one study (Houdaihed et al., 2020). A comprehensive summary of each study and its outcome measures is presented in Table 2.

**TABLE 2:** Characteristics of the included studies.

Study	Drug(s) loaded	Cell line	Purpose	Functionalization
Esim et al. (2024)	Rego + Flu	MCF-7 and MDA-MB-231	Evaluate synergistic anticancer (Rego + Flu) and metabolic modulation effects	Rego-mPEG-b-PLGA
Jafari-Gharabaghlou et al. (2023)	Met	MDA-MB-231	Evaluate anticancer effect via the AMPK pathway and inhibition of the PI3K, Akt, and Ras-MAPK signaling pathways	PEG–PLGA NPs FA-PEG–PLGA
Farajzadeh et al. (2018)	Met + Cur	T47D	Evaluate synergistic apoptosis (Met + Cur) and hTERT inhibition	Met-PEG–PLGA
Yildiz et al. (2018)	Dox	MDA-MB-231	Evaluate chemotherapeutic and imaging theranostic potentials	PLA-PEG-PLL NPs
Haggag et al. (2020)	HK	MCF-7	Evaluate antiproliferative phytochemical effects	PEG–PLGA nanocapsule
El-Hammadi et al. (2017)	5-FU	MCF-7	Compare cytotoxic chemotherapeutic effects	FA-PEG–PLGA
Kordi et al. (2016)	Helenalin	T47D	Evaluate telomerase inhibition effect	PLGA NPs
Singh et al. (2015)	SQV	MCF-7	Compare HIV protease inhibitor with anticancer potential	FA-PEG–PLGA
Houdaihed et al. (2020)	PTX + EVER	MCF-7, MDA-MB-436, and SKBR3	Evaluate dual-targeted combination therapy	PEG-b–PLGA
Sharma et al. (2018)	shRNA-1 and shRNA-4	MDA-MB-231	Evaluate gene silencing of metastasis-related genes	PEG–PLGA NPs
Javan et al. (2019)	Cur + Chr (co-nanoencapsulated)	MDA-MB-231	Evaluate synergistic antiproliferative and apoptotic effects via miRNA regulation	PEG–PLGA NPs
Tabatabaei Mirakabad et al. (2016)	Cur	MCF-7	Compare cytotoxicity between pure curcumin and nanoparticle formulations	PEG–PLGA NPs
Gorjikhah et al. (2017)	Methotrexate	T47D	Compare cytotoxicity and enhanced solubility of methotrexate through nanoparticle encapsulation	PLGA-BC NPs

Chr, chrysin; Cur, curcumin; Dox, doxorubicin; EVER, everolimus; FA, folic acid; Flu, 5-fluorouracil; HK, honokiol; Met, metformin; PEG, poly(ethylene glycol); PLA, poly(lactic acid); PLGA, poly(lactic-co-glycolic acid); PLL, poly(L-lysine); PTX, paclitaxel; Rego, regorafenib; shRNA, short hairpin RNA; SQV, saquinavir; BC, β-cyclodextrin.

### 3.3 Quality evaluation

The quality evaluation results of the studies are summarized in Table 3; most of the studies demonstrated moderate-to-high methodological quality, particularly in terms of randomization, blinding of the *in vitro* replicates, and reproducibility of assays. None of the studies were excluded on quality grounds.

**TABLE 3:** National Institute for Health and Care Excellence methodology checklist: quantitative studies.

Reference	Study design	Population			Method of allocation to intervention (or comparison)										Outcome						Analysis						Summary	
		1	2	3	4	5	6	7	8	9	10	11	12	13	14	15	16	17	18	19	20	21	22	23	24	25	26	27
Esim et al. (2024)	Experimental	++	+	++	NR	++	NA	NA	++	++	NR	NR	NA	NA	++	++	++	++	NR	NR	NA	NA	NR	NR	++	++	++	++
Jafari-Gharabaghlou et al. (2023)	Experimental	++	+	+	NR	++	NA	NA	++	++	NR	NR	NA	NA	++	++	++	++	NR	NR	NA	NA	NR	NR	++	+	+	+
Farajzadeh et al. (2018)	Experimental	++	+	+	NR	++	NA	NA	++	++	NR	NR	NA	NA	++	++	++	++	NR	NR	NA	NA	NR	NR	++	+	++	++
Yildiz et al. (2018)	Experimental	+	+	+	NR	++	NA	NA	++	++	NR	NR	NA	NA	++	++	++	++	NR	NR	NA	NA	NR	NR	++	+	++	++
Haggag et al. (2020)	Experimental	++	+	+	NR	++	NA	NA	++	++	NR	NR	NA	NA	++	++	++	++	NR	+	NA	NA	NR	NR	++	++	++	++
El-Hammadi et al. (2017)	Experimental	++	+	+	NR	++	NA	NA	++	++	NR	NR	NA	NA	++	++	++	++	NR	NR	NA	NA	NR	+	++	++	++	+
Kordi et al. (2016)	Experimental	++	+	+	NA	++	NA	NA	++	++	NR	NR	NA	NA	++	++	++	++	NR	NR	NA	NA	NR	NR	++	+	++	+
Singh et al. (2015)	Experimental	+	+	+	NR	++	NA	NA	++	++	NR	NR	NA	NA	++	+	+	++	NR	NR	NA	NA	NR	NR	++	+	+	+
Houdaihed et al. (2020)	Experimental	+	+	+	NA	++	NA	NA	+	++	++	NA	NA	NA	++	++	++	++	NR	NR	NR	NA	NR	+	+	+	+	+
Sharma et al. (2018)	Experimental	+	+	+	NA	++	NA	NA	++	+	++	NA	NA	NA	++	+	++	++	++	++	+	NA	NR	+	++	+	+	+
Javan et al. (2019)	Experimental	++	+	+	NR	++	NA	NA	+	+	++	NA	NA	NA	+	+	+	++	NR	NR	NR	NA	NR	+	+	+	+	+
Tabatabaei Mirakabad et al. (2016)	Experimental	+	+	+	NR	++	NA	NA	+	+	++	NA	NA	NA	++	+	+	++	+	+	NR	NA	NR	+	+	+	+	+
Gorjikhah et al. (2017)	Experimental	++	+	+	NR	++	NA	NA	+	+	++	NA	NA	NA	++	+	+	+	NR	NR	NR	NA	NR	+	+	+	+	+

Key to headings: Population: 1. Is the source population or source area well described? 2. Is the eligible population or area representative of the source population or area? 3. Do the selected participants or areas represent the eligible populations or areas? Method of allocation to intervention (or comparison): 4. Allocation to intervention (or comparison). How was the selection bias minimized? 5. Were the interventions (and comparisons) well-described and appropriate? 6. Was the allocation concealed? 7. Were the participants or investigators blinded to exposure and comparison? 8. Was the exposure to the intervention and comparison adequate? 9. Was contamination acceptable now? 10. Were the other interventions similar to both groups? 11. Were all participants accounted for at study conclusion? 12. Did the setting reflect usual practices in the UK? 13. Did the intervention or control comparison reflect usual practices in the UK? Outcomes: 14. Were the outcome measures reliable? 15. Were all outcome measurements complete? 16. Were all important outcomes assessed? 17. Were the outcomes relevant? 18. Were there similar follow-up times in both exposure and comparison groups? 19. Was the follow-up time meaningful? Analyses: 20. Were the exposure and comparison groups similar at baseline? If not, were these adjusted? 21. Was the intention to treat (ITT) analysis conducted? 22. Was the study sufficiently powered to detect an intervention effect (if one exists)? 23. Were the estimates of effect size given or calculable? 24. Were the analytical methods appropriate? 25. Was the precision of the intervention effects given or calculable? Were they meaningful? Summary: 26. Are the study results internally valid (i.e., unbiased)? 27. Are the findings generalizable to the source population (i.e., externally valid)? (National Institute for Health and Care Excellence (NICE) methodology checklist: quantitative studies; https://www.nice.org.uk/process/pmg4/chapter/appendix-f-quality-appraisal-checklist-quantitative-intervention-studies, accessed on 1 August 2025). Not applicable (NA) indicates study design aspects that are not applicable given the study design under review; Not reported (NR) indicates aspects in which the study under review fails to report how they have (or may have) been considered; − indicates aspects of the study design in which significant sources of bias may persist; + indicates that either the answer to the checklist question is not clear from the way the study is reported or that the study may not have addressed all potential sources of bias for that particular aspect of the study design; ++ indicates that for that particular aspect of the study design, the study has been designed or conducted in such a way as to minimize the risk of bias.

### 3.4 NP design, synthesis, and characterization

A series of polymeric NPs was successfully synthesized using PLGA and its PEGylated derivatives (PEG–PLGA, mPEG-b-PLGA, and folate-functionalized PEG–PLGA) as carrier matrices (Esim et al., 2024; Jafari-Gharabaghlou et al., 2023; Farajzadeh et al., 2018; Yildiz et al., 2018; Haggag et al., 2020; El-Hammadi et al., 2017; Kordi et al., 2016; Singh et al., 2015; Houdaihed et al., 2020; Sharma et al., 2018; Javan et al., 2019; Tabatabaei Mirakabad et al., 2016; Gorjikhah et al., 2017). The most common fabrication techniques included nanoprecipitation and double-emulsion solvent evaporation (W_1_/O/W_2_), which enabled high encapsulation efficiencies (EEs) and narrow particle size distributions (Jafari-Gharabaghlou et al., 2023; Farajzadeh et al., 2018; Haggag et al., 2020; Kordi et al., 2016; Sharma et al., 2018; Javan et al., 2019). Table 4 shows the comparative physicochemical data for these PEG–PLGA NPs. The formulations were optimized to enhance the solubilities, bioavailabilities, and tumor-targeting potentials of hydrophobic anticancer agents such as regorafenib (Esim et al., 2024), metformin (Jafari-Gharabaghlou et al., 2023; Farajzadeh et al., 2018), curcumin (Javan et al., 2019; Tabatabaei Mirakabad et al., 2016), paclitaxel (Houdaihed et al., 2020), everolimus (Houdaihed et al., 2020), saquinavir (Singh et al., 2015), and honokiol (Haggag et al., 2020), in addition to nucleic acid cargos such as shRNA (Sharma et al., 2018). PEGylation markedly improved the colloidal stability and prolonged circulation, while folate or antibody conjugation enhanced active targeting to folate-receptor-positive or HER2/EGFR-expressing breast cancer cells (Houdaihed et al., 2020). The co-loading strategy (regorafenib + fluorouracil; metformin + curcumin; paclitaxel + everolimus) was noted to achieve synergistic therapeutic ratios while reducing the drug concentrations required for cytotoxicity, confirming the advantages of combination-loaded PEG–PLGA nanosystems (Esim et al., 2024; Farajzadeh et al., 2018; Houdaihed et al., 2020). The zeta-potential measurements showed negative surface charges (in millivolts), which were attributed to the deprotonated carboxyl groups of PLGA (Esim et al., 2024; Jafari-Gharabaghlou et al., 2023; Farajzadeh et al., 2018; Yildiz et al., 2018; Haggag et al., 2020; El-Hammadi et al., 2017; Kordi et al., 2016; Singh et al., 2015; Houdaihed et al., 2020; Sharma et al., 2018; Javan et al., 2019; Tabatabaei Mirakabad et al., 2016; Gorjikhah et al., 2017). This negative potential reduced NP aggregation and prolonged suspension stability under physiological conditions.

**TABLE 4:** Comparative physicochemical data for PEG–PLGA NPs.

Study	Size (mm)	PDI	Zeta potential (mV)	Encapsulation efficiency (%)	Loading capacity (%)
Esim et al. (2024)	Rego-mPEG-b-PLGA: 213.8 ± 16.5 Rego + Flu-mPEG-b-PLGA: 225.5 ± 7.3	Rego-mPEG-b-PLGA: 0.023 Rego + Flu-mPEG-b-PLGA: 0.038	Rego-mPEG-b-PLGA: −21.7 Rego + Flu-mPEG-b-PLGA: −24.3	Rego-mPEG-b-PLGA: 88.7 ± 2.5 Rego + Flu-mPEG-b-PLGA: 32.6 ± 3.6	NR
Jafari-Gharabaghlou et al. (2023)	PEG–PLGA: 176 ± 3.7 Met–PEG–PLGA: 242 ± 6.3 Met–FA–PEG–PLGA: 269 ± 4.4	PEG–PLGA: 0.147 Met–PEG–PLGA: 0.133 Met–FA–PEG–PLGA: 0.098	PEG–PLGA: −29.6 ± 2.1 Met–PEG–PLGA: −32.1 ± 4.6 Met–FA–PEG–PLGA: −33.6 ± 3.5	Met–PEG–PLGA: 64.3 Met–FA–PEG–PLGA: 83.5	Met–PEG–PLGA: 7.3 ± 2.5 Met–FA–PEG–PLGA: 13.7 ± 2.9
Farajzadeh et al. (2018)	PEG–PLGA: 202.8 ± 9.2 Met–PEG–PLGA: 209 ± 10.56 Cur–PEG–PLGA: 223 ± 10.43 Met–Cur–PEG–PLGA: 257 ± 11.38	PEG–PLGA: 0.176 Met–PEG–PLGA: 0.166 Cur–PEG–PLGA: 0.132 Met–Cur–PEG–PLGA: 0.112	PEG–PLGA: −7.23 ± 0.43 Met–PEG–PLGA: −6.1 ± 2.2 Cur–PEG–PLGA: −5.7 ± 2.4 Met–Cur–PEG–PLGA: −3.2 ± 1.2	Met–PEG–PLGA: 75.15 Cur–PEG–PLGA: 80.5	Met–PEG–PLGA: 12.5 ± 2.1 Cur–PEG–PLGA: 10.1 ± 2.2
Yildiz et al. (2018)	Blank NPs: 60.0 ± 11.7 Dox-FB NPs: 62.5 ± 10.7 Dox-FB-AF750: 78.8 ± 21.5	Blank NPs: 0.33 ± 0.12 Dox-FB NPs: 0.22 ± 0.06 Dox-FB-AF750: 0.22 ± 0.09	Blank NPs: −9.2 ± 6.1 Dox-FB NPs: −11.2 ± 6.6 Dox-FB-AF750: −6.9 ± 7.8	NR	NR
Haggag et al. (2020)	HK-5%PEG–PLGA: 216.52 ± 15.95 HK-10%PEG–PLGA: 167.51 ± 14.43 HK-15%PEG–PLGA: 125.12 ± 11.51	HK-5%PEG–PLGA: 0.21 ± 0.05 HK-10%PEG–PLGA: 0.28 ± 0.04 HK-15%PEG–PLGA: 0.19 ± 0.06	HK-5%PEG–PLGA: −9.97 ± 2.81 HK-10%PEG–PLGA: −8.11 ± 1.04 HK-15%PEG–PLGA: −6.21 ± 0.97	HK-5%PEG–PLGA: 73 ± 4.5 HK-10%PEG–PLGA: 80 ± 2.0 HK-15%PEG–PLGA: 90 ± 1.97	NR
El-Hammadi et al. (2017)	PEG–PLGA: 145.3 ± 10.4 FA-PEG–PLGA: 149.2 ± 7.0	PEG-PLGA: 0.066 ± 10.4 FA-PEG–PLGA: 0.075 ± 0.059	PEG–PLGA: −22.3 ± 2.1 FA-PEG–PLGA: −15.7 ± 1.4	PEG–PLGA: 8.73 ± 0.44 FA-PEG–PLGA: 8.68 ± 0.51	PEG–PLGA: 5.52 ± 0.14 FA-PEG–PLGA: 5.49 ± 0.15
Kordi et al. (2016)	PEG_10_–PLGA: 77 PEG_30_–PLGA: 55	NR	PEG–PLGA: −33.2	PEG_10_–PLGA: 64.2 ± 3.5 PEG_30_–PLGA: 72.3 ± 4.6	
Singh et al. (2015)	SQV-PLGA: 118.8 ± 2.52 SQV-FA–PEG–PLGA: 223.8 ± 5.42	SQV-PLGA: 0.15 ± 0.002 SQV-FA–PEG–PLGA: 0.18 ± 0.005	SQV–PLGA: −27.961 ± 3.43 SQV-FA–PEG–PLGA: −8.967 ± 5.09	SQV-PLGA: 56.00 ± 0.68 SQV-FA–PEG–PLGA: 58.00 ± 0.80	NR
Houdaihed et al. (2020)	PTX + EVER + UT-NPs: 80.9 ± 6.5 PTX + EVER + T-NPs: 98.7 ± 4.2 PTX + EVER + Dual-NPs: 104.5 ± 5.3	NR	PTX + EVER + UT-NPs: −7.5 ± 2.3 PTX + EVER + T-NPs: −7.9 ± 2.7 PTX + EVER + Dual-NPs: −9.2 ± 3.3	PTX + EVER + UT-NPs: 46.6 ± 1.6 PTX + EVER + T-NPs: 40.9 ± 4.9 PTX + EVER + Dual-NPs: 39.6 ± 1.5	PTX + EVER + UT-NPs: 6.5 ± 0.2 PTX + EVER + T-NPs: 5.8 ± 0.6 PTX + EVER + Dual-NPs: 5.6 ± 0.2
Sharma et al. (2018)	PLGA-shRNA-1: 426.41 ± 26.23 PEG-5%–PLGA-shRNA-1: 251.61 ± 14.71 PEG-10%–PLGA-shRNA-1: 272.36 ± 9.82 PLGA-shRNA-4: 408.45 ± 20.18 PEG-5%–PLGA-shRNA-4: 289.93 ± 13.24 PEG-10%–PLGA- shRNA-4: 237.71 ± 23.36	PLGA-shRNA-1: 0.414 ± 0.144 PEG-5%–PLGA-shRNA-1: 0.231 ± 0.015 PEG-10%–PLGA-shRNA-1: 0.336 ± 0.018 PLGA-shRNA-4: 0.348 ± 0.012 PEG-5%–PLGA-shRNA-4: 0.201 ± 0.020 PEG-10%–PLGA- shRNA-4: 0.265 ± 0.011	PLGA-shRNA-1: −0.332 ± 0.66 PEG-5%–PLGA-shRNA-1: −0.224 ± 1.03 PEG-10%–PLGA-shRNA-1: −0.092 ± 0.37 PLGA-shRNA-4: −2.71 ± 0.039 PEG-5%–PLGA-shRNA-4: −1.81 ± 0.023 PEG-10%–PLGA- shRNA-4: −1.17 ± 0.044	PLGA-shRNA-1: 74.94 ± 1.32 PEG-5%–PLGA-shRNA-1: 75.10 ± 7.67 PEG-10%–PLGA-shRNA-1: 66.31 ± 3.74 PLGA-shRNA-4: 85.18 ± 2.52 PEG-5%–PLGA-shRNA-4: 84.02 ± 5.81 PEG-10%–PLGA- shRNA-4: 80.01 ± 1.06	PLGA-shRNA-1 (in µg/mg): 0.10 ± 0.01 PEG-5%–PLGA-shRNA-1 (in µg/mg): 0.10 ± 0.10 PEG-10%–PLGA-shRNA-1 (in µg/mg): 0.09 ± 0.05 PLGA-shRNA-4 (in µg/mg): 0.11 ± 0.03 PEG-5%–PLGA-shRNA-4 (in µg/mg): 0.11 ± 0.08 PEG-10%–PLGA-shRNA-4 (in µg/mg): 0.11 ± 0.02
Javan et al. (2019)	PEG–PLGA: 200 ± 4.5 Cur–PEG–PLGA: 234 ± 7.45 Chr–PEG–PLGA: 233 ± 10.30 Cur/Chr–PEG–PLGA: 305 ± 5.45	PEG–PLGA: 0.165 Cur–PEG–PLGA: 0.151 Chr–PEG–PLGA: 0.148 Cur/Chr–PEG–PLGA: 0.120	PEG–PLGA: −7.5 ± 2.6 Cur–PEG–PLGA: −6.5 ± 2.1 Chr–PEG–PLGA: −6.3 ± 3.5 Cur/Chr–PEG–PLGA: −3.8 ± 3.1	Cur–PEG–PLGA: 80.22 Chr–PEG–PLGA: 85.25 Cur/Chr–PEG–PLGA: 68.5	Cur–PEG–PLGA: 13.5 ± 3.5 Chr–PEG–PLGA: 12.3 ± 2.2 Cur/Chr–PEG–PLGA: 10.86 ± 3.5
Tabatabaei Mirakabad et al. (2016)	Cur-PEG-PLGA: 70–300	NR	NR	Cur–PEG–PLGA: 84.5	NR
Gorjikhah et al. (2017)	MTX-PLGA-BC: 70–200	NR	NR	MTX-PLGA–BC: 80	MTX-PLGA–BC: 18

AF750, Alexa Fluor 750 (fluorescent dye); BC, β-cyclodextrin; Chr, chrysin; Cur, curcumin; Dox, doxorubicin; EVER, everolimus; FA, folic acid; FB, fluorobenzyl; Flu, 5-fluorouracil; HK, honokiol; Met, metformin; MTX, methotrexate; NR, not reported; PDI, polydispersity index; PEG, poly(ethylene glycol); PLA, poly(lactic acid); PLGA, poly(lactic-co-glycolic acid); PLL, poly(L-lysine); PTX, paclitaxel; Rego, regorafenib; shRNA, short hairpin RNA; SQV, saquinavir; UT-NPs, untargeted nanoparticles; T-NPs, HER2-targeted nanoparticles; Dual-NPs: dual-targeted nanoparticles.

### 3.5 Cytotoxicity, cellular uptake, apoptosis, and molecular mechanisms

Controlled drug release was most commonly evaluated using dialysis-based assays (Esim et al., 2024; Jafari-Gharabaghlou et al., 2023; Yildiz et al., 2018; Haggag et al., 2020; Singh et al., 2015; Javan et al., 2019) or high-performance liquid chromatography (HPLC) (Houdaihed et al., 2020), with all studies reporting sustained-release patterns. A summary of the outcomes is shown in Table 5. The cellular uptake was visualized via fluorescence microscopy, HPLC, and flow cytometry (Esim et al., 2024; Jafari-Gharabaghlou et al., 2023; Yildiz et al., 2018; Haggag et al., 2020; Houdaihed et al., 2020; Javan et al., 2019). PEGylation and folate targeting significantly enhanced the internalization and cytoplasmic localization of NPs without nuclear entry (Jafari-Gharabaghlou et al., 2023; Singh et al., 2015; Houdaihed et al., 2020; Sharma et al., 2018). Esim et al. (2024) demonstrated increased intracellular accumulation of regorafenib when encapsulated in NPs, particularly in the MDA-MB-231 cells; however, fluorouracil uptake remained unchanged, likely owing to differences in P-glycoprotein (P-gp) expression. Similarly, Jafari-Gharabaghlou et al. (2023) reported that metformin-loaded folate-PEG–PLGA NPs had higher cellular uptake than non-targeted PEG–PLGA formulations. In the MDA-MB-231 cells, Haggag et al. (2020) found that optimal uptake occurred for NPs composed of 10% PEGylated PLGA. In contrast, Sharma et al. (2018) observed that coumarin-6-labeled NPs were localized exclusively in the cytoplasm of the MDA-MB-231 cells without nuclear penetration. Javan et al. (2019) further indicated that NP internalization occurred predominantly through endocytic pathways. In the MCF-7 cells, Singh et al. (2015) showed that folate-targeted PEG–PLGA NPs encapsulating saquinavir (SQV-FA-PEG–PLGA) exhibited greater fluorescence intensity than either free saquinavir or non-targeted SQV-PLGA NPs.

**TABLE 5:** Summary of cytotoxicity outcomes.

Study	Exposure time	IC_50_ (μM)	% cell death	Comparison vs. free drug
Esim et al. (2024)	24–72 h	Rego-mPEG-b-PLGA: 24.6 ± 12.1 (MCF-7 at 72 h); 34.5 ± >20 (MDA-MB-231 at 72 h) Rego + Flu-mPEG-b-PLGA: 4.7 ± 3.0 (MCF-7 at 72 h); 6.4 ± 1.4 (MDA-MB-231 at 72 h)	Late apoptosis: 6.38% (MCF-7), 10.98% (MDA-MB-231), strong synergy (CI < 1, DRI ≈ 5–13× Rego reduction)	Combination NPs required 5.34–12.83× less Rego + 6.78–35× less Flu to achieve same inhibition; enhanced cytotoxicity vs. free drugs and monotherapies
Jafari-Gharabaghlou et al. (2023)	48 h	Met–PEG–PLGA: 10.34 Met–FA–PEG–PLGA: 7.89	Cell survival ↓ to 75% (Met), 40% (Met-PEG-PLGA), 21% (Met-FA-PEG-PLGA)	Folate targeting increased uptake (54.6% vs. 39.8%) and enhanced cytotoxicity (∼2× vs. Met-PEG-PLGA, ∼3× vs. free Met)
Farajzadeh et al. (2018)	24–72 h	Met–PEG–PLGA: 8.71 (72 h) Cur–PEG–PLGA: 8.87 (72 h) Met–Cur–PEG–PLGA: 4.76 (72 h)	Synergistic inhibition of hTERT; viability ↓ > 70% (Met-Cur-PEG-PLGA)	Combination > single drugs
Yildiz et al. (2018)	72 h	NR	Significant cell viability ↓ (dose-dependent)	Sustained 30-day release; enhanced imaging
Haggag et al. (2020)	24–72 h	HK-5%PEG–PLGA: 50 ± 1.3 (72 h) HK-10%PEG–PLGA: 35 ± 2.3 (72 h) HK-15%PEG–PLGA: 20 ± 2.3 (72 h)	Strong dose-dependent cytotoxicity; uptake 71% ± 6.9%	PEGylation ↑ uptake; blank NPs non-toxic
El-Hammadi et al. (2017)	72 h	PEG–PLGA: 7.68 ± 0.53 FA-PEG–PLGA: 3.92 ± 0.42	IC_50_ ↓ 4× vs. free 5-FU	PEGylation ↑ uptake, folate-receptor-mediated endocytosis, overcoming P-gp efflux
Kordi et al. (2016)	24–72 h	Helenalin: 1.3 (72 h) Helenalin-PEG_10_–PLGA: 0.16 (48 h) Helenalin-PEG_10_–PLGA: 0.14 (72 h)	9× higher potency vs. free helenalin	9× higher potency vs. free helenalin
Singh et al. (2015)	24–72 h	NR	12%–15% inhibition (260 μM, MCF-7)	Higher inhibition vs. PLGA or free drug
Houdaihed et al. (2020)	24–96 h	PTX + EVER + UT-NPs: 34.8 ± 6.2 (SKBR3); 43.8 ± 3.1 (MCF-7) PTX + EVER + T-NPs: 20.6 ± 4.3 (SKBR3); 41.5 ± 2.1 (MCF-7) PTX + EVER + Dual-NPs: 5.6 ± 0.2 (SKBR3); 33.8 ± 6.1 (MCF-7)	Cytotoxicity ↑ vs. monotargeted and untargeted drugs	Dual-NPs > T-NPs; free drug comparable after 4 days
Sharma et al. (2018)	24–96 h	NR	Cell viability ↓ significantly vs. controls	Encapsulation → > 80% efficiency; free shRNA ineffective
Javan et al. (2019)	48 h	Cur: 20.23 Chr: 60.03 Cur–PEG–PLGA: 31.55 Chr–PEG–PLGA: 69.18 Cur/Chr: 14.14 (Cur) and 28.28 (Chr) Cur/Chr–PEG–PLGA: 9.988 (Cur) and 19.98 (Chr)	Cur/Chr-NPs showed highest cytotoxicity; ↑ apoptosis and G2/M arrest	Synergistic effect > free Cur + Chr
Tabatabaei Mirakabad et al. (2016)	24–72 h	Cur: 31.14 ± 1.24 (24 h); 21.32 ± 1.61 (48 h); 11.31 ± 1.47 (72 h) Cur–PEG–PLGA: 17.86 ± 1.08 (24 h); 16.31 ± 1.31 (48 h); 6.21 ± 1.18 (72 h)	25 µM (NP) vs. 45 μM (free Cur) in MCF-7	Cur-NPs > free Cur
Gorjikhah et al. (2017)	24 h	MTX (in mg/mL): 0.391 (24 h); 0.361 (48 h); 0.285 (72 h) MTX-PLGA–BC (in mg/mL): 0.318 (24 h); 0.294 (48 h); 0.241 (72 h)	20 µM (MTX-NP) vs. 35 μM (free MTX) in T47D	NPs > free MTX

AMPK, AMP-activated protein kinase; BC, β-cyclodextrin; Chr, chrysin; CI, combination index; Cur, curcumin; DRI, dose reduction index; EVER, everolimus; FA, folic acid; FB, fluorobenzyl; Flu, 5-fluorouracil; hTERT, human telomerase reverse transcriptase; HK, honokiol; IC_50_, half maximal inhibitory concentration; Met, metformin; MTX, methotrexate; NPs, nanoparticles; NR, not reported; PEG, poly(ethylene glycol); PI3K, phosphoinositide 3-kinase; PLA, poly(lactic acid); PLGA, poly(lactic-co-glycolic acid); PLL, poly(L-lysine); P-gp, P-glycoprotein; PTX, paclitaxel; Rego, regorafenib; shRNA, short hairpin RNA; SKBR3, HER2-positive breast cancer cell line; SQV, saquinavir; T-NPs, HER2-targeted nanoparticles; Dual-NPs, dual-targeted nanoparticles; UT-NPs, untargeted nanoparticles.

The apoptotic effects were mostly verified by flow cytometry and reverse transcription polymerase chain reaction (RT-PCR) (Esim et al., 2024; Jafari-Gharabaghlou et al., 2023; Farajzadeh et al., 2018; Haggag et al., 2020; Kordi et al., 2016; Javan et al., 2019). Esim et al. (2024) showed that the regorafenib +fluorouracil-mPEG-b-PLGA formulation produced greater cytotoxicity than either regorafenib alone or rego@mPEG-b-PLGA in both the MCF-7 and MDA-MB-231 cell lines. In the MDA-MB-231 cells, Jafari-Gharabaghlou et al. (2023) demonstrated that metformin-loaded FA-PEG–PLGA NPs significantly upregulated proapoptotic genes such as p53, Bax, and caspases while downregulating antiapoptotic markers such as Bcl-2 and hTERT. Similarly, Farajzadeh et al. (2018) found that metformin–curcumin-loaded NPs induced cell-cycle arrest in the T47D cells and promoted Bax and caspase expressions, whereas both free metformin–curcumin and metformin–curcumin-PLGA/PEG NPs decreased hTERT mRNA expression in a dose-dependent manner. In the MDA-MB-231 cells, Haggag et al. (2020) reported that peptide-functionalized NPs caused cell-cycle arrest at the G0/G1 and G2/M phases, while Kordi et al. (2016) observed that NC-helenalin NPs markedly reduced hTERT expression compared to free helenalin. Sharma et al. (2018) noted a pronounced reduction in MDA-MB-231 cell viability following treatment with 10% PEG–PLGA NPs. Among the included studies, the highest apoptosis rate was recorded in MDA-MB-231 cells treated with curcumin–chrysin co-loaded NPs (Javan et al., 2019).

### 3.6 Additional assessments

Several studies incorporated complementary analyses to further characterize the biological effects of the PEG–PLGA NP formulations. These included metabolomics (Esim et al., 2024), Ran-GTP expression (Haggag et al., 2020), and blood compatibility testing (El-Hammadi et al., 2017). Haggag et al. (2020) demonstrated that peptide-functionalized NPs not only reduced breast cancer cell viability but also suppressed the expression of Ran-GTP, which is a protein associated with tumor growth and progression. In addition, El-Hammadi et al. (2017) confirmed the biocompatibility of their system and reported no cytotoxic effects in normal human cell lines, including CCD-18 colon fibroblasts and MCF-10A non-tumorigenic breast epithelial cells.

## 4 Discussion

### 4.1 Summary of key findings and interpretation

This systematic review of 13 experimental studies highlights the promising roles of PEG–PLGA NPs as drug-delivery systems for antitumor agents in the treatment of breast cancer. Across all the cell models examined in these studies (MCF-7, MDA-MB-231, and T47D), the nanocarriers consistently improved drug encapsulation, sustained release, and cytotoxicity compared to the free formulations. Formulations with optimal physicochemical properties (150–250 nm size, polydispersity index (PDI) <0.2, zeta potential (ζ) = −20 to −35 mV) were reported to achieve the strongest antiproliferative effects, confirming the relevance of nanoscale stability in biological performance. For example, regorafenib/fluorouracil-mPEG-b-PLGA NPs (225.5 ± 7.3 nm, ζ = −24.3 mV, EE = 32.6%) showed late apoptosis rates of 6.38% (MCF-7) and 10.98% (MDA-MB-231), reducing IC_50_ from 24.6 to 4.7 µM and allowing 5–13 fewer regorafenib and 6–35 fewer fluorouracil doses (Esim et al., 2024). Furthermore, metformin-PEG–PLGA NPs (242 ± 6 nm, ζ = −32.1 mV, EE = 64%) and folate-modified variants (269 ± 4 nm, ζ = −33.6 mV, EE = 83.5%) decreased cell survival to 40% and 21%, respectively, confirming enhanced folate receptor uptake and cytotoxicity by nearly thrice those of free metformin (Jafari-Gharabaghlou et al., 2023). Similarly, metformin/curcumin–PEG–PLGA (257 ± 11 nm, ζ = −3.2 mV) was reported to induce >70% viability loss with synergistic hTERT inhibition, outperforming single-drug systems (Farajzadeh et al., 2018). Natural-compound-based NPs comprising honokiol, curcumin, chrysin, and helenalin showed EEs of 80%–90% and up to 9-fold greater cytotoxicity than free agents (Haggag et al., 2020; El-Hammadi et al., 2017; Kordi et al., 2016; Javan et al., 2019; Tabatabaei Mirakabad et al., 2016). Even large systems such as shRNA-PLGA (237–426 nm, EE ∼80%) were noted to achieve gene silencing and reduce invasiveness in the MDA-MB-231 cells (Sharma et al., 2018). These consistent outcomes underline that PEG–PLGA nanocarriers can potentiate drug performance through improved solubility, controlled release, and receptor-mediated uptake.

### 4.2 Mechanistic determinants of enhanced efficacy

Certain formulations such as co-loaded or folate-functionalized PEG–PLGA NPs can achieve superior performance through both physicochemical and biological mechanisms. For example, regorafenib/fluorouracil dual-loaded NPs (rego/flu-mPEG-b-PLGA) achieved a dose reduction of up to 12 times with increased late apoptosis (6.38% in MCF-7 and 10.98% in MDA-MB-231), which are attributable to combined inhibition of the glycolytic and tricarboxylic acid cycle pathways (Esim et al., 2024). Similarly, folate-functionalized metformin-loaded PEG–PLGA NPs induced strong downregulation of *hTERT* and *Bcl-2* while upregulating *Bax*, *Caspase-3/7*, and *p53* (Jafari-Gharabaghlou et al., 2023), consistent with the activation of mitochondrial apoptosis through modulation of the AMPK/mTOR axis. These observations reveal that improved cytotoxicity is not merely a consequence of the higher intracellular drug concentration but a sign of coordinated metabolic and transcriptional reprogramming triggered by nanodelivery. Moreover, PEG–PLGA NPs have been repeatedly shown to prolong circulation time, reduce immunogenicity, and enhance targeting when functionalized with ligands such as folic acid and peptides (Gao et al., 2023; Liu et al., 2021a; Liu et al., 2021b; Liu et al., 2019; Liu et al., 2022).

Targeted uptake, PEGylation effects, and intracellular signaling modulation are all considered to increase the efficacy. Dual-targeted paclitaxel + everolimus NPs (104 ± 5 nm) showed the best selectivity in HER2^high and EGFR^mod cells, lowering the IC_50_ value to 5.6 µM from 34.8 µM for constructs that were not targeted (Houdaihed et al., 2020). In addition, folate functionalization significantly increased internalization (54.6% vs. 39.8% for non-targeted NPs) and nearly doubled the cytotoxicity (Jafari-Gharabaghlou et al., 2023), as observed in formulations containing 5-fluorouracil (El-Hammadi et al., 2017), saquinavir (Singh et al., 2015), and metformin (Jafari-Gharabaghlou et al., 2023). These improvements are owed to the folate-receptor-mediated endocytosis, which selectively boosts the uptake of NPs in breast tumor cells that have too many folate receptors. Furthermore, PEGylation further prolongs NP circulation and minimizes premature opsonization, producing smaller particle sizes (typically 100–220 nm) and narrower PDIs (*p* < 0.05) that are characteristics known to favor the enhanced permeability and retention (EPR) effect.

At the molecular level, several formulations activated intrinsic apoptosis via the p53/Bax/Caspase-3/7 pathway and inhibited the Bcl-2, PI3K/Akt/mTOR, and Ras-MAPK pathways (Jafari-Gharabaghlou et al., 2023; Farajzadeh et al., 2018; Kordi et al., 2016). Metabolomic analyses demonstrated the inhibition of glycolysis and tricarboxylic acid intermediates subsequent to co-delivery of regorafenib and fluorouracil (Esim et al., 2024), suggesting metabolic reprogramming as a cytotoxic mechanism. Curcumin–chrysin NPs were reported to inhibit NF-κB/p65 and HN1 while enhancing miR-132 and miR-502c, establishing a connection between nanoencapsulation and epigenetic remodeling (Javan et al., 2019). Similarly, shRNA-PLGA systems achieved *RAN* gene knockdown, thereby limiting metastasis-related signaling (Sharma et al., 2018). Collectively, these findings demonstrate that PEG–PLGA nanocarriers act through combined physical targeting and molecular pathway modulation.

### 4.3 Patterns of cytotoxicity and apoptosis

Across the studies considered herein, PEG–PLGA NPs produced consistent dose-dependent cytotoxic effects with significant reductions in the IC_50_ values compared to free drugs. For example, metformin/curcumin–PLGA-PEG NPs achieved synergistic growth inhibition and greater suppression of hTERT expression than either agent alone (Farajzadeh et al., 2018); honokiol-loaded PEG–PLGA nanocapsules produced up to 80% growth inhibition in MCF-7 cells and significantly reduced the tumor volume *in vivo* (Haggag et al., 2020). The integration of apoptosis-related assays (MTT, Annexin-V/PI, and qPCR) corroborated these cytotoxic findings, highlighting the roles of nanodelivery systems in restoring apoptotic sensitivity in resistant phenotypes like the triple-negative MDA-MB-231. The data in Tables 2, 4, 5 indicate well-defined structure–activity relationships among the PEG–PLGA NP formulations. A decrease in particle size was consistently associated with enhanced cellular uptake and cytotoxicity; for instance, honokiol NPs of size 125 nm exhibited an IC_50_ value of 20 μM, whereas larger particles of size 216 nm reached 50 µM (Haggag et al., 2020). Moderately negative surface charges (ζ = −25 to −35 mV) enhanced the colloidal stability and facilitated electrostatic interactions with negatively charged cellular membranes (Esim et al., 2024; Jafari-Gharabaghlou et al., 2023). High EEs (>75%), such as those reported for curcumin (Tabatabaei Mirakabad et al., 2016), methotrexate (Gorjikhah et al., 2017), and phytochemical formulations (Javan et al., 2019), were linked to prolonged intracellular drug retention and sustained therapeutic levels. Most of these systems displayed a biphasic release pattern characterized by an initial ≈20% burst, followed by 48–96 h of controlled release, thereby maintaining effective cytotoxic concentrations over time (Farajzadeh et al., 2018; Haggag et al., 2020). These findings indicate that the most effective formulations possess ideal physicochemical profiles characterized by particle sizes ranging from 150 to 250 nm, PDIs less than 0.2, zeta potentials between −25 and −35 mV, and EEs exceeding 75% (Esim et al., 2024; Jafari-Gharabaghlou et al., 2023; Farajzadeh et al., 2018; Yildiz et al., 2018; Haggag et al., 2020; El-Hammadi et al., 2017; Kordi et al., 2016; Singh et al., 2015; Houdaihed et al., 2020; Sharma et al., 2018; Javan et al., 2019; Tabatabaei Mirakabad et al., 2016; Gorjikhah et al., 2017).

The cytotoxic performances of the PEG–PLGA formulations demonstrated marked improvements over the corresponding free drugs, with enhancements ranging from 2-fold to 12-fold depending on the active compound and targeting strategy. For example, rego/flu-mPEG-b-PLGA NPs achieved an IC_50_ value of 4.7 µM versus 24.6 µM for the free combination, accompanied by 6%–11% increase in the apoptotic activity (Esim et al., 2024). Metformin functionalized with folic acid (met-FA-PEG–PLGA) showed an IC_50_ value of 7.9 µM compared to >20 µM for the unencapsulated drug, corresponding to a three-fold increase in cytotoxicity (Jafari-Gharabaghlou et al., 2023). Similarly, metformin–curcumin–PEG–PLGA NPs reached an IC_50_ value of 4.8 µM versus >8 μM for the free formulations, reducing cell viability by approximately 70% (Farajzadeh et al., 2018). Helenalin–PEG–PLGA showed the largest improvement, with an IC_50_ value of 0.14 µM compared to 1.3 µM for the free drug, amounting to nine-fold better efficacy (Kordi et al., 2016). Dual-loaded curcumin/chrysin–PEG–PLGA NPs (IC_50_ ≈ 10 µM) exhibited synergistic cytotoxic effects that surpassed those of the free drug mixture (Javan et al., 2019). Finally, MTX-PLGA-β-CD NPs ensured better solubility of methotrexate that was more toxic to the cells, which lowered the IC_50_ value from 35 μM to 20 µM (Gorjikhah et al., 2017). Collectively, these results confirm that PEG–PLGA encapsulation enhances drug solubility, stability, and cellular penetration while effectively lowering the required systemic dose for comparable or superior therapeutic outcomes.

### 4.4 Critical synthesis and future directions

The collective findings of this survey highlight a coherent mechanistic framework underlying the therapeutic performances of PEG–PLGA nanocarriers. Encapsulation within the PEG–PLGA matrix significantly enhances drug solubility and promotes tumoral accumulation via the EPR effect (Esim et al., 2024; Jafari-Gharabaghlou et al., 2023; Farajzadeh et al., 2018; Yildiz et al., 2018; Haggag et al., 2020; El-Hammadi et al., 2017; Kordi et al., 2016; Singh et al., 2015; Houdaihed et al., 2020; Sharma et al., 2018; Javan et al., 2019; Tabatabaei Mirakabad et al., 2016; Gorjikhah et al., 2017). Surface modification through PEGylation and ligand conjugation further optimize the cellular uptake and receptor-mediated selectivity, thereby improving the targeting efficiency (Jafari-Gharabaghlou et al., 2023; Farajzadeh et al., 2018; El-Hammadi et al., 2017). Co-encapsulation strategies like regorafenib + fluorouracil, metformin + curcumin, and curcumin + chrysin produce synergistic activation of the metabolic and apoptotic pathways, thereby amplifying the anticancer efficacies beyond monotherapy outcomes (Esim et al., 2024; Farajzadeh et al., 2018; Javan et al., 2019). Additionally, modulation of the gene and miRNA expressions can broaden the therapeutic spectra of these nanoplatforms (Sharma et al., 2018; Javan et al., 2019), while theranostic formulations like doxorubicin-loaded NPs integrate imaging capabilities with chemotherapeutic action to support real-time treatment monitoring (Yildiz et al., 2018). Previous studies have described the properties of doxorubicin-loaded folate-biotin NPs (Dox-FB) as an initial burst release on the first day followed by a prolonged and controlled release phase (Yusuf et al., 2023; Lee et al., 2008), while peptide-loaded NPs reportedly maintained their activity against MDA-MB-231 cells for up to 4 days (Samec et al., 2022). To advance clinical translation of these findings, future studies should consider incorporating 3D tumor spheroid models, biodistribution and pharmacokinetic mapping, as well as development of good-manufacturing-practice-compliant PEG–PLGA prototypes to ensure scalability, reproducibility, and regulatory readiness for human trials.

### 4.5 Scope and limitations

This systematic review demonstrates several strengths that reinforce its scientific value. The inclusion of studies from diverse geographic regions, encompassing multiple breast cancer cell lines and drug types, enhances the robustness and generalizability of the findings. The methodologies employed in these studies were comprehensive, integrating physicochemical characterizations, cytotoxicity assays, mechanistic analyses, and safety assessments, thereby providing a holistic understanding of the performance of NP systems. Comparative approaches, such as ligand functionalization and dual-drug-delivery strategies, were also reported in some cases, offering additional insights into therapeutic optimization. Furthermore, mechanistic evaluations using advanced techniques such as flow cytometry, RT-PCR, and metabolomic profiling enriched the analyses and strengthened the evidence base. Irrespective of these strengths, several limitations must be acknowledged. The reliance on *in vitro* models without complementary *in vivo* or clinical investigations can be considered a restriction to the translational applicability of these findings. Considerable heterogeneities in the drug dosages, NP compositions, and experimental protocols also complicate direct comparisons while limiting the feasibility of meta-analytic integration. Some critical pharmacological parameters such as biodistribution, clearance, and inflammatory responses were rarely assessed in the studies, leaving a gap in the understanding of the systemic behaviors of these formulations. Moreover, the absence of real-time imaging approaches precluded evaluation of any theranostic applications or stimulus-responsive release mechanisms. Future studies should therefore emphasize *in vivo* preclinical investigations that address the pharmacokinetics, biodistribution, and tumor-specific accumulation under physiologically relevant conditions. Expanding the research efforts into theranostics and remotely triggered PLGA-based platforms could further accelerate the clinical translation of these findings to breast cancer therapy.

## 5 Conclusion

This systematic review indicates that polymeric NPs, especially those composed of PEG–PLGA as the loading matrix, could serve as highly efficient vehicles for the targeted delivery of antineoplastic drugs in breast cancer models. These nanoformulations, when optimized to a size of 150–250 nm, zeta potential of approximately −25 to −35 mV, and EE > 75%, can consistently demonstrate superior performances relative to their free drug formulations, including enhanced cellular uptake, prolonged drug release, increased cytotoxic effects, and modulation of the apoptosis-related pathways. The incorporation of targeted ligands such as folic acid can enhance therapeutic selectivity, highlighting the potential to mitigate the drawbacks of traditional chemotherapy, such as chemoresistance and off-target damage. Through its convergent mechanisms, PEG–PLGA constitutes a clinically promising nanoplatform for multidrug and multitarget breast cancer therapy that aligns bioengineering control with translational oncology goals.

## Data Availability

The original contributions presented in the study are included in the article/Supplementary Material; further inquiries can be directed to the corresponding authors.

## References

[B1] Cardoso F. Senkus E. Costa A. Papadopoulos E. Aapro M. André F. (2020). 4th ESO-ESMO international consensus guidelines for advanced breast cancer (ABC 4). Ann. Oncol. 31, 1623–1649. 10.1016/j.annonc.2020.09.010 30032243 PMC7360146

[B2] Colditz G. A. Bohlke K. (2014). Priorities for the primary prevention of breast cancer. CA Cancer J. Clin. 64, 186–194. 10.3322/caac.21225 24647877

[B3] Danhier F. Ansorena E. Silva J. M. Coco R. Le Breton A. Préat V. (2012). PLGA-based nanoparticles: an overview of biomedical applications. J. Control. Release 161, 505–522. 10.1016/j.jconrel.2012.01.043 22353619

[B4] D’Orsi C. J. Bassett L. W. Berg W. A. Feig S. A. Jackson V. P. Kopans D. B. (2013). Breast imaging reporting and data system: ACR BI-RADS—Breast imaging Atlas. 4th ed. Reston: American College of Radiology.

[B5] El-Hammadi M. M. Delgado Á. V. Melguizo C. Prados J. C. Arias J. L. (2017). Folic acid-decorated and PEGylated PLGA nanoparticles for improving the antitumour activity of 5-fluorouracil. Int. J. Pharm. 516 (1-2), 61–70. 10.1016/j.ijpharm.2016.11.012 27825867

[B6] Esim O. Adatepe S. Sarper M. Bakirhan N. K. Erdoğan Kablan S. Kocak E. (2024). The potential role of hot flashes treatment strategies and regorafenib combinations in breast cancer therapy via co-drug loaded polymeric nanoparticles. J. Drug Deliv. Sci. Technol. 98, 105898. 10.1016/j.jddst.2024.105898

[B7] Farajzadeh R. Pilehvar-Soltanahmadi Y. Dadashpour M. Javidfar S. Lotfi-Attari J. Sadeghzadeh H. (2018). Nano-encapsulated metformin-curcumin in PLGA/PEG inhibits synergistically growth and hTERT gene expression in human breast cancer cells. Artif. Cells Nanomed. Biotechnol. 46 (5), 917–925. 10.1080/21691401.2017.1347879 28678551

[B8] Fessi H. Puisieux F. Devissaguet J. P. Ammoury N. Benita S. (1989). Nanocapsule formation by interfacial polymer deposition following solvent displacement. Int. J. Pharm. 55, R1–R4. 10.1016/0378-5173(89)90281-0

[B9] Gao Y. Wang K. Zhang J. Duan X. Sun Q. Men K. (2023). Multifunctional nanoparticle for cancer therapy. MedComm 4 (1), e187. 10.1002/mco2.187 36654533 PMC9834710

[B10] Gorjikhah F. Azizi Jalalian F. Salehi R. Panahi Y. Hasanzadeh A. Alizadeh E. (2017). Preparation and characterization of PLGA-β-CD polymeric nanoparticles containing methotrexate and evaluation of their effects on T47D cell line. Artif. Cells Nanomed. Biotechnol. 45 (3), 432–440. 10.3109/21691401.2016.1160915 27002986

[B11] Haggag Y. A. Ibrahim R. R. Hafiz A. A. (2020). Design, formulation and *in vivo* evaluation of novel honokiol-loaded PEGylated PLGA nanocapsules for treatment of breast cancer. Int. J. Nanomed. 15, 1625–1642. 10.2147/IJN.S241428 32210557 PMC7069567

[B12] Houdaihed L. Evans J. C. Allen C. (2020). Dual-targeted delivery of nanoparticles encapsulating paclitaxel and everolimus: a novel strategy to overcome breast cancer receptor heterogeneity. Pharm. Res. 37 (3), 39. 10.1007/s11095-019-2684-6 31965330

[B13] Jafari-Gharabaghlou D. Dadashpour M. Joodi Khanghah O. Salmani-Javan E. Zarghami N. (2023). Potentiation of folate-functionalized PLGA-PEG nanoparticles loaded with metformin for the treatment of breast cancer: possible clinical application. Mol. Biol. Rep. 50 (4), 3023–3033. 10.1007/s11033-022-08171-w 36662452

[B14] Javan N. Khadem Ansari M. H. Dadashpour M. Khojastehfard M. Bastami M. Rahmati-Yamchi M. (2019). Synergistic antiproliferative effects of co-nanoencapsulated curcumin and chrysin on MDA-MB-231 breast cancer cells through upregulating miR-132 and miR-502c. Nutr. Cancer 71 (7), 1201–1213. 10.1080/01635581.2019.1599968 30955355

[B15] Jokerst J. V. Lobovkina T. Zare R. N. Gambhir S. S. (2011). Nanoparticle PEGylation for imaging and therapy. Nanomedicine (London) 6, 715–728. 10.2217/nnm.11.19 21718180 PMC3217316

[B16] Key T. J. Verkasalo P. K. Banks E. (2001). Epidemiology of breast cancer. Lancet Oncol. 2, 133–140. 10.1016/S1470-2045(00)00254-0 11902563

[B17] Kordi S. Zarghami N. Akbarzadeh A. Rahmati Y. M. Ghasemali S. Barkhordari A. (2016). A comparison of the inhibitory effect of nano-encapsulated helenalin and free helenalin on telomerase gene expression in the breast cancer cell line, by real-time PCR. Artif. Cells Nanomed. Biotechnol. 44 (2), 695–703. 10.3109/21691401.2014.981270 25435410

[B18] Lakhani S. R. Ellis I. O. Schnitt S. J. Tan P. H. van de Vijver M. J. (2012). WHO classification of tumours of the breast. 4th ed. (Lyon: IARC).

[B19] Lee E. S. Gao Z. Kim D. Park K. Kwon I. C. Bae Y. H. (2008). Super pH-sensitive multifunctional polymeric micelle for tumor pH(e) specific TAT exposure and multidrug resistance. J. Control. Release 129, 228–236. 10.1016/j.jconrel.2008.04.024 18539355 PMC2603624

[B20] Liu M. Wang B. Guo C. Hou X. Cheng Z. Chen D. (2019). Novel multifunctional triple folic acid, biotin and CD44 targeting pH-sensitive nano-actiniaes for breast cancer combinational therapy. Drug Deliv. 26 (1), 1002–1016. 10.1080/10717544.2019.1669734 31571501 PMC6781222

[B21] Liu J. Ai X. Cabral H. Liu J. Huang Y. Mi P. (2021a). Tumor hypoxia-activated combinatorial nanomedicine triggers systemic antitumor immunity to effectively eradicate advanced breast cancer. Biomaterials 273, 120847. 10.1016/j.biomaterials.2021.120847 33932702

[B22] Liu J. Tian B. Liu Y. Wan J. B. Suhovskih A. V. Kazanskaya G. M. (2021b). Cyclodextrin-containing hydrogels: a review of preparation method, drug delivery, and degradation behavior. Int. J. Mol. Sci. 22 (24), 13350. 10.3390/ijms222413350 34948312 PMC8703588

[B23] Liu Y. Wang L. Song Q. Ali M. Crowe W. N. Kucera G. L. (2022). Intrapleural nano-immunotherapy promotes innate and adaptive immune responses to enhance anti-PD-L1 therapy for malignant pleural effusion. Nat. Nanotechnol. 17, 206–216. 10.1038/s41565-021-01032-w 34916656 PMC9074399

[B24] Makadia H. K. Siegel S. J. (2011). Poly lactic-co-glycolic acid (PLGA) as biodegradable controlled drug delivery carrier. Polymers (Basel) 3, 1377–1397. 10.3390/polym3031377 22577513 PMC3347861

[B25] Mu L. Feng S. S. (2003). A novel controlled release formulation for the anticancer drug paclitaxel (Taxol®): PLGA nanoparticles containing vitamin E TPGS. J. Control. Release 86, 33–48. 10.1016/S0168-3659(02)00320-6 12490371

[B26] National Institute for Health and Care Excellence (2012). “Appendix F: quality appraisal checklist—Quantitative intervention studies,” in Methods for the development of NICE public health guidance (London, UK: National Institute for Health and Care Excellence). Available online at: https://www.nice.org.uk/process/pmg4/chapter/about-this-document (Accessed August 1, 2025). 27905711

[B27] Owens D. E. Peppas N. A. (2006). Opsonization, biodistribution, and pharmacokinetics of polymeric nanoparticles. Int. J. Pharm. 307, 93–102. 10.1016/j.ijpharm.2005.10.010 16303268

[B28] Page M. J. McKenzie J. E. Bossuyt P. M. Boutron I. Hoffmann T. C. Mulrow C. D. (2021). PRISMA 2020 statement: an updated guideline for the publication of systematic reviews. BMJ 74, 790–799. 10.1136/bmj.n71 33781348 PMC8008539

[B29] Parveen S. Misra R. Sahoo S. K. (2012). Nanoparticles: a boon to drug delivery, therapeutics, diagnostics and imaging. Nanomedicine 8, 147–166. 10.1016/j.nano.2011.05.016 21703993

[B30] Samec T. Boulos J. Gilmore S. Hazelton A. Alexander-Bryant A. (2022). Peptide-based delivery of therapeutics in cancer treatment. Mater. Today Bio. 14, 100248. 10.1016/j.mtbio.2022.100248 35434595 PMC9010702

[B31] Schnitt S. J. (2010). Classification and prognosis of invasive breast cancer: from morphology to molecular taxonomy. Mod. Pathol. 23, S60–S64. 10.1038/modpathol.2010.33 20436504

[B32] Sharma A. McCarron P. Matchett K. Hawthorne S. El-Tanani M. (2018). Anti-invasive and anti-proliferative effects of shRNA-loaded poly(lactide-co-glycolide) nanoparticles following RAN silencing in MDA-MB231 breast cancer cells. Pharm. Res. 36 (2), 26. 10.1007/s11095-018-2555-6 30560466 PMC6297200

[B33] Shi J. Votruba A. R. Farokhzad O. C. Langer R. (2010). Nanotechnology in drug delivery and tissue engineering: from discovery to applications. Nano Lett. 10, 3223–3230. 10.1021/nl102184c 20726522 PMC2935937

[B34] Singh R. Kesharwani P. Mehra N. K. Singh S. Banerjee S. Jain N. K. (2015). Development and characterization of folate-anchored saquinavir entrapped PLGA nanoparticles for anti-tumor activity. Drug Dev. Ind. Pharm. 41 (11), 1888–1901. 10.3109/03639045.2015.1019355 25738812

[B35] Sung H. Ferlay J. Siegel R. L. Laversanne M. Soerjomataram I. Jemal A. (2021). Global cancer statistics 2020: GLOBOCAN estimates of incidence and mortality worldwide for 36 cancers in 185 countries. CA Cancer J. Clin. 71, 209–249. 10.3322/caac.21660 33538338

[B36] Tabatabaei Mirakabad F. S. Akbarzadeh A. Milani M. Zarghami N. Taheri-Anganeh M. Zeighamian V. (2016). A comparison between the cytotoxic effects of pure curcumin and curcumin-loaded PLGA-PEG nanoparticles on the MCF-7 human breast cancer cell line. Artif. Cells Nanomed. Biotechnol. 44 (1), 423–430. 10.3109/21691401.2014.955108 25229832

[B37] Vásquez E. Sandoval C. (2025). Effectiveness of drug-loaded PEG-PLGA nanoparticles in cancer treatment: a systematic review. PROSPERO CRD420251076570. Available online at: https://www.crd.york.ac.uk/PROSPERO/view/CRD420251076570.

[B38] Yildiz T. Gu R. Zauscher S. Betancourt T. (2018). Doxorubicin-loaded protease-activated near-infrared fluorescent polymeric nanoparticles for imaging and therapy of cancer. Int. J. Nanomed. 13, 6961–6986. 10.2147/IJN.S174068 30464453 PMC6217908

[B39] Yusuf A. Almotairy A. R. Z. Henidi H. Alshehri O. Y. Aldughaim M. S. (2023). Nanoparticles as drug delivery systems: a review of the implication of nanoparticles' physicochemical properties on responses in biological systems. Polymers 15, 1596. 10.3390/polym15071596 37050210 PMC10096782

